# Prognostic significance of the advanced lung cancer inflammation index for long-term mortality in patients with acute myocardial infarction and diabetes

**DOI:** 10.3389/fendo.2026.1876399

**Published:** 2026-07-06

**Authors:** Hongqiang Li, Jiachen Luo, Gang Li, Fei Yang, Yidong Wei

**Affiliations:** 1 General Intensive Care Unit (ICU), The First Affiliated Hospital of Zhengzhou University, Zhengzhou, China; 2 Department of Cardiology, Shanghai Tenth People’s Hospital, Tongji University, Shanghai, China; 3 Department of Cardiology, The First Affiliated Hospital of Zhengzhou University, Zhengzhou, China

**Keywords:** acute myocardial infarction, advanced lung cancer inflammation index (ALI), diabetes, mortality, prognosis

## Abstract

**Background:**

The advanced Lung Cancer Inflammation Index (ALI) is a novel composite marker integrating nutritional, metabolic, and inflammatory status. This study aimed to investigate the prognostic significance of admission ALI for long-term mortality in patients with acute myocardial infarction (AMI) and concomitant diabetes.

**Methods:**

A total of 571 diabetic AMI patients were retrospectively enrolled from the NOAFCAMI-SH registry. Patients were stratified into ALI tertiles (T1-T3). The primary and secondary outcomes were all-cause and cardiovascular mortality, respectively, evaluated over a median follow-up of 2.5 years. Associations were assessed using Kaplan-Meier survival curves, Cox proportional hazards regression, and restricted cubic spline (RCS) models.

**Results:**

Compared to the lowest tertile (T1), patients in the highest tertile (T3) exhibited markedly lower rates of all-cause (7.9% vs. 27.8%) and cardiovascular mortality (7.4% vs. 23.6%). In fully adjusted Cox models, higher ALI (T3 vs. T1) was independently associated with a 67% reduction in the risk of all-cause mortality (HR = 0.33, 95% CI: 0.18–0.61, P < 0.001) and a similar significant decrease in cardiovascular mortality. RCS analysis revealed a significant non-linear, L-shaped relationship between continuous ALI and both mortality outcomes (both P for non-linearity < 0.05), with mortality risk rising precipitously below a specific threshold. Furthermore, adding ALI to the established GRACE score modestly improved individual-level risk reclassification and discrimination for all-cause mortality (continuous NRI: 0.228, P = 0.047; IDI: 0.027, P = 0.020).

**Conclusion:**

A lower admission ALI independently associated with long-term all-cause and cardiovascular mortality in diabetic AMI patients.As a readily accessible composite biomarker, ALI offers valuable prognostic information that could assist in the early risk stratification of this high-risk population.

## Introduction

Acute myocardial infarction (AMI) ranks among the foremost contributors to cardiovascular-related death worldwide, with systemic inflammation driving its pathogenesis (1, 2). Diabetes is a prevalent comorbidity that exacerbates AMI outcomes, as both conditions share inflammation as a key pathophysiological factor (3, 4). Chronic low-grade inflammation in diabetic patients accelerates atherosclerosis and impairs myocardial repair (5). Therefore, identifying biomarkers reflecting this inflammation-metabolism interplay is critical for refining risk assessment strategies in AMI patients with diabetes.

Composite inflammatory indices, such as the Systemic Immune-Inflammation Index (SII) and Neutrophil-to-Lymphocyte Ratio (NLR), independently predict adverse AMI outcomes (6–8). Notably, our prior research demonstrated that a high SII specifically predicts long-term mortality in AMI patients with DM, but not in non-diabetics (9). This highlights that the prognostic value of inflammatory markers is amplified within the specific metabolic context of diabetes.

The advanced Lung Cancer Inflammation Index (ALI) is a novel composite marker integrating nutritional and inflammatory status (10). Beyond its oncologic origins, ALI has shown prognostic utility across cardiovascular conditions, including acute coronary syndrome, heart failure, and myocardial infarction with nonobstructive coronary arteries(MINOCA) (11–13). Lower ALI consistently correlates with increased all-cause and cardiovascular mortality (14). Furthermore, in populations with metabolic disorders like type 2 diabetes, a significant inverse association exists between ALI and mortality (15).

Despite its value in broader cardiovascular populations, no study has investigated ALI’s prognostic significance specifically in the high-risk cohort of AMI patients with DM. Given the profound inflammatory and metabolic disturbances characterizing this comorbidity (16), we hypothesize that ALI—which uniquely integrates inflammation, nutrition, and metabolism—could serve as a powerful mortality risk predictor for this vulnerable population.

Therefore, this study aims to investigate the association between admission ALI levels and long-term all-cause and cardiovascular mortality in diabetic AMI patients using a clinical registry database. We anticipate this readily available biomarker will enhance risk stratification and personalized management for this cohort.

## Materials and methods

### Study design and population

This investigation was conducted as a retrospective observational study drawing on data from the NOAFCAMI-SH registry (ClinicalTrials.gov Identifier: NCT03533543). We initially screened 2,399 consecutive patients diagnosed with AMI and admitted to the Cardiac Care Unit (CCU) of Shanghai Tenth People’s Hospital between February 2014 and March 2018. The diagnosis of AMI was established in strict accordance with the Fourth Universal Definition of Myocardial Infarction.

To focus specifically on the diabetic AMI population and ensure data completeness for the calculation of the ALI, candidates were systematically excluded on the basis of the following criteria: (1) absence of diabetes; (2) age younger than 18 years; (3) loss to follow-up; (4) absence of recorded body mass index (BMI) (BMI) data; and (5) missing laboratory data required for ALI calculation (including admission levels of albumin, neutrophils, or lymphocytes).

**Figure 1:**
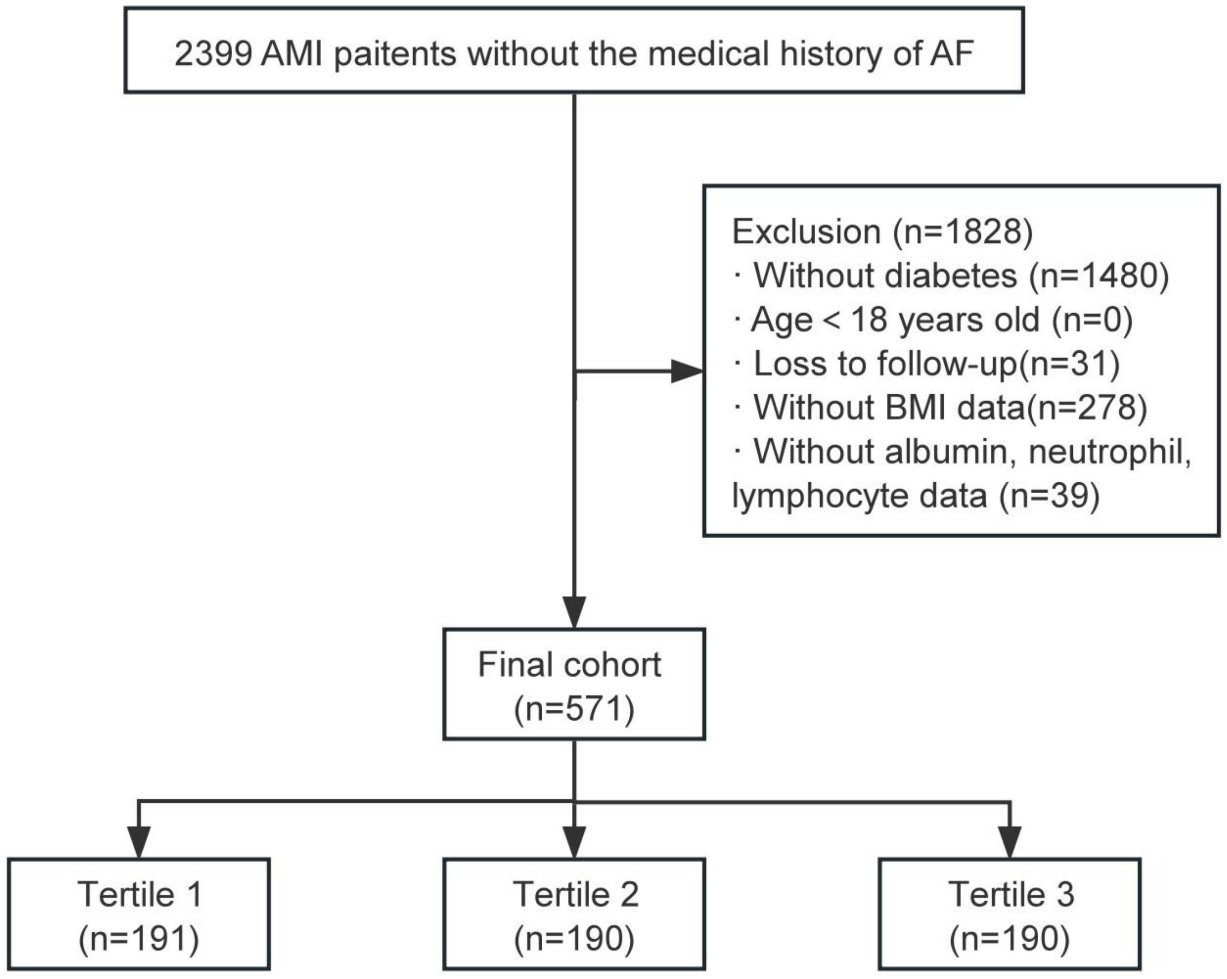
Flowchart of patient selection and cohort stratification.

Following the application of these exclusion conditions, a final cohort of 571 diabetic AMI patients was included in the present analysis. The stepwise patient selection procedure is illustrated in **Figure 1**. The study protocol complied with the ethical principles outlined in the Declaration of Helsinki and received formal approval from the Ethics Committee of Shanghai Tenth People’s Hospital (Approval No. SHSY-IEC-KY-4.1/18–199/01).Since all information was anonymized during extraction, the requirement for informed consent was exempted.

### Data collection

Demographic, clinical, and laboratory information were retrospectively retrieved from electronic medical records. Fasting venous blood was drawn within 24 hours of admission and analyzed at the central laboratory. ALI was calculated as: ALI = BMI (kg/m²) × albumin (g/dL)/(neutrophils/lymphocytes). Patients were stratified into ALI tertiles: T1 (≤ 15.20), T2 (15.20–29.89), and T3 (> 29.89).

Covariates included demographics (age, sex, BMI, smoking), admission vitals (heart rate, systolic blood pressure, Killip class), infarction type, and comorbidities (hypertension, dyslipidemia, chronic kidney disease, prior heart failure, stroke, or myocardial infarction). We also collected echocardiographic parameters (LAD, LVEDD, LVESD, LVEF) and routine laboratory indices (blood counts, lipid profile, glucose, HbA1c, creatinine, eGFR, C-reactive protein, and peak troponin T). Treatment variables comprised percutaneous coronary intervention (PCI) and medications (aspirin, statins, ACEI/ARB, and β-blockers).

### Outcomes

Enrolled patients were followed for a median duration of 2.5 years.Survival status and causes of death were independently adjudicated by two experienced cardiologists through a blinded review of medical records and follow-up documentation.The principal study endpoint was all-cause mortality, while the secondary endpoint was cardiovascular mortality. Cardiovascular death was defined as death resulting from cardiovascular causes, including acute myocardial infarction, heart failure, or malignant arrhythmias. In accordance with standard epidemiological practice, deaths were classified as cardiovascular unless a definite non-cardiovascular cause was clearly documented.

### Statistical analysis

Continuous parameters were expressed as means accompanied by standard deviations (SD) and compared using analysis of variance (ANOVA) if normally distributed. Categorical variables are presented as frequencies with percentages and analyzed using the Chi-square test. Missing values for clinical variables accounted for less than 5% of the total data and were imputed using the median of the respective variables. To explore the relationships between ALI and key clinical parameters (peak cardiac troponin T, CRP, and LVEF), generalized additive models (GAM) equipped with smoothing splines were employed. Survival probabilities across the distinct ALI strata were estimated using the Kaplan-Meier estimator, with between-group divergences evaluated via the log-rank test. We used sequentially adjusted Cox models to assess the prognostic value of ALI on mortality, verifying the proportional hazards assumption using Schoenfeld residuals.The selection of covariates was driven by clinical relevance, established prognostic factors reported in the myocardial infarction literature, and LASSO regression analysis(**Supplementary Figure 1**), with the results of the univariable analysis detailed in **Supplementary Tables 1** and **2**.Before modeling, the variance inflation factor (VIF) was checked to ensure the absence of severe multicollinearity among the covariates. Three sequential models were applied: Model 1 was crude without any adjustment; Model 2 adjusted for age and gender; and Model 3 was fully adjusted for age, gender, current smoking, hypertension, ST-segment elevation myocardial infarction (STEMI), Killip class, percutaneous coronary intervention (PCI), peak troponin T, HbA1c, and creatinine. Additionally, restricted cubic splines were utilized to visually model the dose-response relationship between continuous ALI and mortality risk, with four knots placed at the 5th, 35th, 65th, and 95th percentiles of the ALI distribution, and the median value serving as the reference.To evaluate the incremental predictive value of ALI, we assessed whether adding ALI to the established GRACE score could improve prognostic accuracy for all-cause mortality. Model performance improvement was quantified by calculating the change in Harrell’s C-index, the continuous net reclassification improvement (NRI), and the integrated discrimination improvement (IDI).Exploratory subgroup analyses were performed to determine the consistency of the ALI-mortality association across diverse clinical strata, including age (<65 vs. ≥65 years), gender (male vs. female), hypertension, LVEF, myocardial infarction type (STEMI vs. NSTEMI), and the performance of PCI. Potential effect modifications were evaluated using likelihood ratio tests for interaction terms. Finally, a sensitivity analysis was conducted by further adjusting for medications (aspirin, ACEI/ARBs, β-blockers, and statins) on top of the variables included in Model 3.All the statistical analyses were prespecified and conducted via stata18 and R software (version 4.4.3) A two-sided P value <0.05 was considered statistically significant.

## Results

### Baseline characteristics

**Table 1** summarizes the baseline characteristics of the study population (N = 571) stratified by ALI tertiles. Patients in the highest ALI tertile exhibited a distinct, more favorable clinical profile. They were significantly younger (63.7 ± 11.7 vs. 69.3 ± 11.4 years, P < 0.001), had higher BMI, and presented with more stable conditions upon admission, including a lower incidence of STEMI (47.9% vs. 67.5%) and Killip class > 1 (P < 0.05).

**Table 1:** Baseline characteristics of the study population according to ALI tertiles.

Characteristic	Overall (N = 571)	Tertile 1 (N = 191)	Tertile 2 (N = 190)	Tertile 3 (N = 190)	P-value
Demographics					
Age, years	66.5 ± 12.3	69.3 ± 11.4	66.4 ± 13.2	63.7 ± 11.7	<0.001
Male	422 (73.9)	142 (74.3)	139 (73.2)	141 (74.2)	0.959
BMI, kg/m²	25.0 ± 3.2	23.9 ± 2.8	25.0 ± 3.2	26.0 ± 3.2	<0.001
Current smoker	239 (41.9)	71 (37.2)	78 (41.1)	90 (47.4)	0.126
Heart rate, bpm	83.1 ± 18.2	87.6 ± 19.5	83.0 ± 17.2	78.8 ± 16.8	<0.001
SBP, mmHg	140.1 ± 25.7	135.3 ± 26.1	142.9 ± 25.1	142.2 ± 25.5	0.006
Killip class>1	91 (15.9)	45 (23.6)	22 (11.6)	24 (12.6)	0.002
Comorbidities					
Hypertension	398 (69.7)	135 (70.7)	131 (68.9)	132 (69.5)	0.931
Dyslipidemia	157 (27.5)	50 (26.2)	54 (28.4)	53 (27.9)	0.877
CKD	79 (13.8)	39 (20.4)	26 (13.7)	14 (7.4)	0.001
Previous HF	42 (7.4)	21 (11.0)	10 (5.3)	11 (5.8)	0.060
Previous stroke	82 (14.4)	32 (16.8)	28 (14.7)	22 (11.6)	0.349
Previous MI	42 (7.4)	14 (7.3)	10 (5.3)	18 (9.5)	0.291
STEMI	332 (58.1)	129 (67.5)	112 (58.9)	91 (47.9)	<0.001
PCI	487 (85.3)	157 (82.2)	165 (86.8)	165 (86.8)	0.336
Laboratory					
WBC, ×10^9^/L	10.1 ± 3.7	12.1 ± 4.0	9.8 ± 3.5	8.3 ± 2.3	<0.001
Neutrophils, ×10^9^/L	8.1 ± 7.0	11.8 ± 10.8	7.3 ± 2.4	5.2 ± 1.8	<0.001
Lymphocytes, ×10^9^/L	1.7 ± 1.1	1.1 ± 0.4	1.6 ± 0.5	2.5 ± 1.4	<0.001
RBC, ×10¹²/L	4.5 ± 1.9	4.4 ± 1.0	4.7 ± 3.1	4.5 ± 0.8	0.267
Platelets, ×10^9^/L	235.5 ± 644.2	213.3 ± 82.8	204.2 ± 59.7	289.1 ± 1112.0	0.370
CRP, mg/L	26.2 ± 42.7	43.6 ± 56.0	21.3 ± 37.0	13.5 ± 22.1	<0.001
Albumin, g/dL	3.8 ± 0.4	3.7 ± 0.5	3.9 ± 0.4	3.9 ± 0.4	<0.001
Glucose, mmol/L	9.9 ± 4.3	11.2 ± 4.8	9.5 ± 3.7	8.9 ± 4.1	<0.001
HbA1c, %	8.2 ± 1.7	8.3 ± 1.9	7.9 ± 1.6	8.2 ± 1.7	0.058
TC, mmol/L	4.4 ± 1.2	4.4 ± 1.1	4.4 ± 1.0	4.6 ± 1.5	0.223
TG, mmol/L	2.0 ± 1.7	1.5 ± 1.0	1.9 ± 1.6	2.4 ± 2.2	<0.001
Creatinine, μmol/L	93.1 ± 67.3	110.2 ± 103.4	86.8 ± 35.7	82.3 ± 34.2	<0.001
eGFR, mL/min/1.73m²	77.8 ± 25.9	69.7 ± 27.6	80.0 ± 24.5	83.8 ± 23.3	<0.001
Peak troponin T, ng/mL	4.4 ± 3.8	5.8 ± 3.9	4.2 ± 3.7	3.4 ± 3.5	<0.001
Echocardiographic data					
LAD, mm	35.4 ± 10.8	35.2 ± 11.9	35.6 ± 10.2	35.3 ± 10.4	0.929
LVEDD, mm	45.8 ± 4.4	46.0 ± 4.5	45.4 ± 4.5	45.9 ± 4.1	0.360
LVESD, mm	31.3 ± 5.4	32.4 ± 5.9	30.6 ± 4.9	31.0 ± 5.2	0.003
LVEF, %	47.2 ± 11.0	43.3 ± 11.3	48.7 ± 11.0	49.4 ± 9.5	<0.001
Medications					
Aspirin	544 (95.3)	181 (94.8)	181 (95.3)	182 (95.8)	0.895
Satins	563 (98.6)	186 (97.4)	190 (100.0)	187 (98.4)	0.091
ACEI/ARB	418 (73.2)	129 (67.5)	135 (71.1)	154 (81.1)	0.008
β-blockers	460 (80.6)	148 (77.5)	155 (81.6)	157 (82.6)	0.407

ACEI, Angiotensin-converting enzyme inhibitor; ARB, Angiotensin II receptor blocker; HF, heart failure; CKD, Chronic kidney disease; CRP, C-reactive protein; HBA1C, Glycated hemoglobin; LAD, Left atrial diameter; LVEDD, Left ventricular end-diastolic diameter; LVEF, Left ventricular ejection fraction; LVESD, Left ventricular end-systolic diameter; PCI, Percutaneous coronary intervention; SBP, Systolic blood pressure; STEMI, ST-elevation myocardial infarction; TC, Total cholesterol; TG, Triglycerides.

Laboratory and echocardiographic findings indicated that higher ALI levels strongly correlated with attenuated systemic inflammation and better nutritional status, evidenced by significantly lower WBC, neutrophils, and CRP, alongside higher lymphocytes and albumin (P< 0.001). Moreover, T3 patients demonstrated more preserved renal function (higher eGFR) and less severe myocardial injury, characterized by lower peak troponin T (3.4 ± 3.5 vs. 5.8 ± 3.9 ng/mL) and higher left ventricular ejection fraction (49.4% vs. 43.3%) (P< 0.001).

### Clinical outcomes according to ALI tertiles

**Table 2** demonstrates a consistent inverse relationship between ALI tertiles and adverse outcomes. Compared to T1, T3 patients showed markedly lower rates of all-cause mortality (7.9% vs. 27.8%), cardiac death (7.4% vs. 23.6%), HF readmission (9.5% vs. 22.5%), and recurrent MI (2.6% vs. 7.3%) (P< 0.05). Notably, stroke incidence did not differ significantly across tertiles (P = 0.502), suggesting ALI’s prognostic value may be specific to cardiac rather than cerebrovascular diseases.

**Table 2:** Clinical outcomes of the study population according to ALI tertiles.

Outcomes, n (%)	Overall (N = 571)	Tertile 1 (N = 191)	Tertile 2 (N = 190)	Tertile 3 (N = 190)	P-value
HF readmission	81 (14.2)	43 (22.5)	20 (10.5)	18 (9.5)	<0.001
Recurrent MI	24 (4.2)	14 (7.3)	5 (2.6)	5 (2.6)	0.031
Stroke	18 (3.2)	6 (3.1)	4 (2.1)	8 (4.2)	0.502
All-cause death	91 (15.9)	53 (27.8)	23 (12.2)	15 (7.9)	<0.001
Cardiac death	76 (13.3)	45 (23.6)	17 (9.0)	14 (7.4)	<0.001

### Associations of ALI with inflammation, myocardial injury, and cardiac function

**Figure 2:**
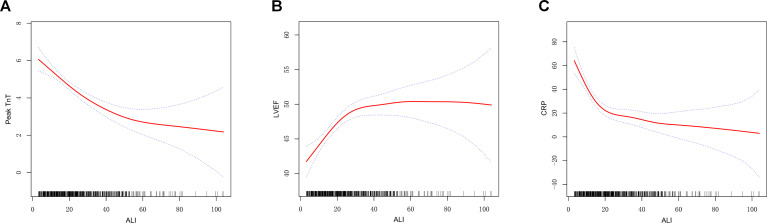
GAM analysis of non-linear associations between ALI and peak troponin T **(A)**, LVEF **(B)**, and CRP **(C)**. Red lines represent fitted smoothing splines; gray shaded areas indicate 95% confidence intervals.

GAM analysis revealed significant non-linear, dose-dependent inverse associations between ALI and CRP and peak troponin T, with effect magnitudes attenuating at higher ALI values. Conversely, ALI demonstrated a significant positive association with LVEF, suggesting preserved cardiac function at higher ALI levels (**Figure 2**).

### Association between ALI and mortality risks

**Figure 3:**
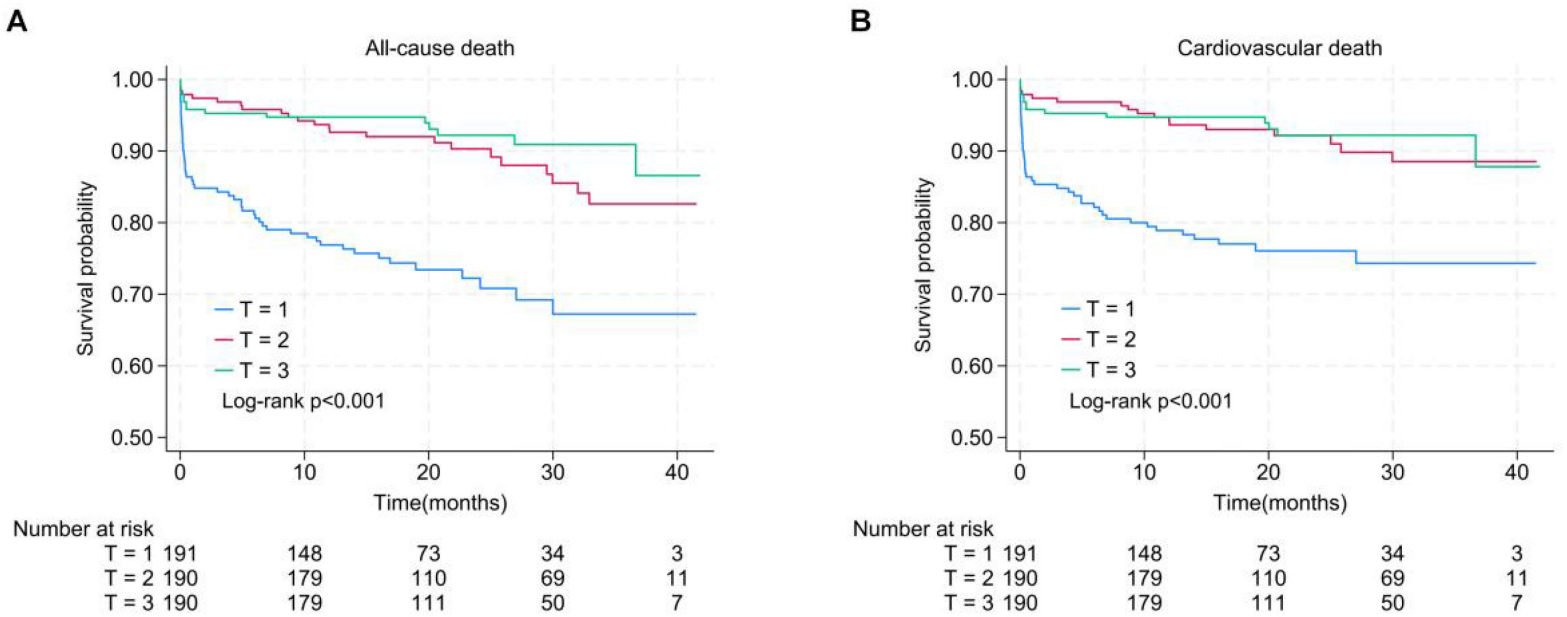
Kaplan-Meier survival curves for all-cause death **(A)** and cardiovascular death **(B)** stratified by ALI tertiles.

The prognostic implications of varying ALI levels were visually corroborated through Kaplan-Meier survival trajectories (**Figure 3**). Over the median 2.5-year tracking period, the survival curves diverged markedly: patients situated in the lowest ALI tertile (T1) experienced drastically inferior survival rates for both all-cause and cardiovascular endpoints when juxtaposed with their counterparts in the mid (T2) and high (T3) tertiles.

**Table 3** illustrates the results of Cox regression models assessing the independent association of ALI with mortality. When ALI was treated as a continuous variable, the protective associations remained robust in the fully adjusted Model 3 for both all-cause mortality (HR = 0.97, 95% CI: 0.95–0.99, P = 0.001) and cardiac mortality (HR = 0.97, 95% CI: 0.96–0.99, P = 0.007). When ALI was categorized into tertiles, a graded inverse association was consistently observed across all models (P for trend < 0.001). In Model 3, compared to T1, T2 and T3 were associated with a 59% (HR = 0.41, 95% CI: 0.24–0.70) and 67% (HR = 0.33, 95% CI: 0.18–0.61) reduction in the risk of all-cause mortality, respectively. Similarly, a significant reduction in the risk of cardiac mortality was observed for T2 (HR = 0.37, 95% CI: 0.21–0.67) and T3 (HR = 0.40, 95% CI: 0.21–0.76) (P < 0.01).

**Table 3:** Association between ALI with all-cause and cardiac death.

Variable	Model 1	P-value	Model 2	P-value	Model 3	P-value
	HR (95%CI)		HR (95%CI)		HR (95%CI)	
All-cause death						
ALI	0.96 (0.94, 0.97)	<0.001	0.96 (0.94, 0.98)	<0.001	0.97 (0.95, 0.99)	0.001
ALI tertiles						
T1	1.00 (Ref)		1.00 (Ref)		1.00 (Ref)	
T2	0.35 (0.22, 0.58)	<0.001	0.36 (0.22, 0.60)	<0.001	0.41 (0.24, 0.70)	0.001
T3	0.24 (0.13, 0.42)	<0.001	0.29 (0.16, 0.53)	<0.001	0.33 (0.18, 0.61)	<0.001
P for trend	0.46 (0.34, 0.61)	<0.001	0.50 (0.37, 0.67)	<0.001	0.54 (0.39, 0.74)	<0.001
Cardiac death						
ALI	0.96 (0.94, 0.98)	<0.001	0.96 (0.94, 0.98)	<0.001	0.97 (0.96, 0.99)	0.007
ALI tertiles						
T1	1.00 (Ref)		1.00 (Ref)		1.00 (Ref)	
T2	0.32 (0.18, 0.56)	<0.001	0.33 (0.19, 0.58)	<0.001	0.37 (0.21, 0.67)	0.001
T3	0.27 (0.15, 0.49)	<0.001	0.33 (0.18, 0.61)	<0.001	0.40 (0.21, 0.76)	0.006
P for trend	0.47 (0.35, 0.65)	<0.001	0.51 (0.37, 0.71)	<0.001	0.58 (0.41, 0.81)	0.001

Model 1: Unadjusted.

Model 2: Adjusted for age and gender.

Model 3: Adjusted for age, gender, current smoking, hypertension, STEMI, Killip class, PCI, peak TnT, HbA1c, creatinine.

**Figure 4:**
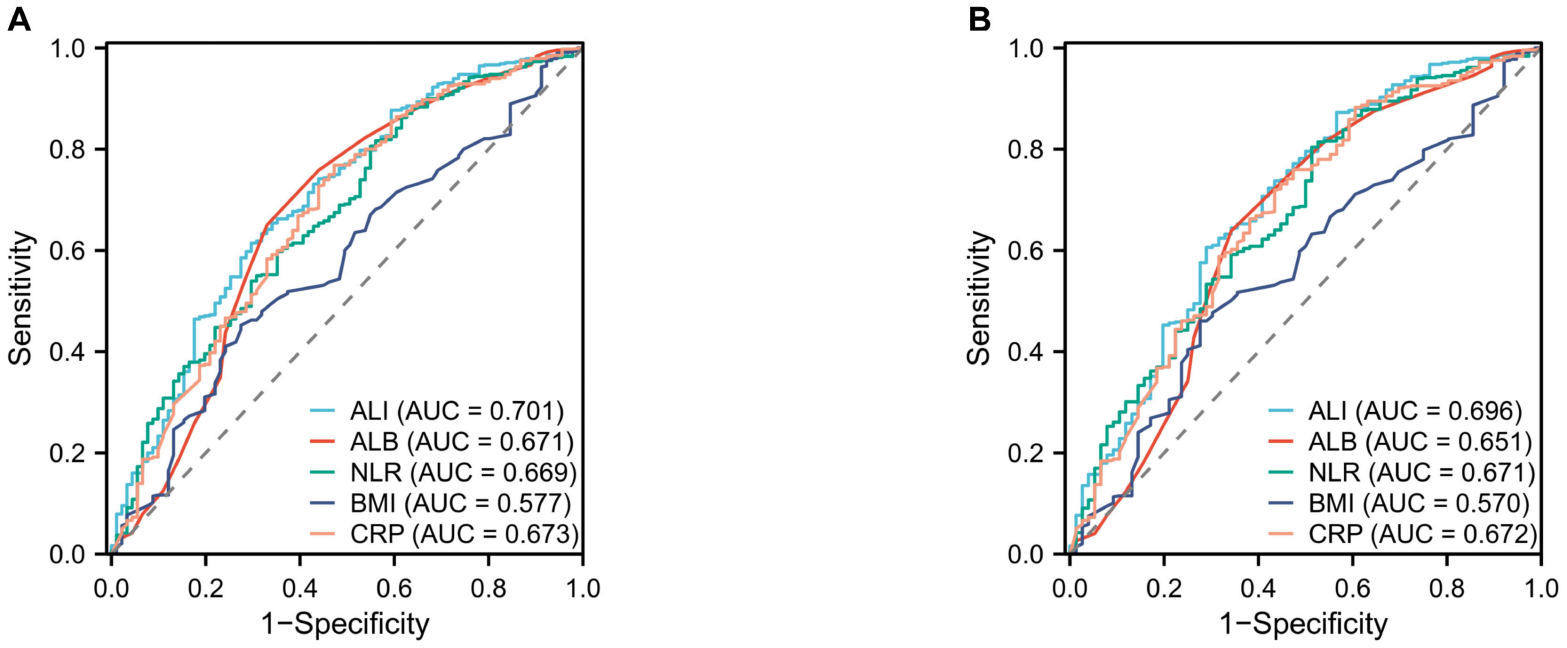
ROC curves for for all-cause mortality **(A)** and cardiovascular mortality **(B)**.

To compare ALI and NLR’s predictive performance for mortality, ROC curve analyses were conducted for all-cause and cardiovascular mortality. ALI showed a significantly higher AUC than NLR for both all-cause (0.701 vs. 0.669, P < 0.001; **Figure 4A**) and cardiovascular mortality (0.696 vs. 0.671, P = 0.005; **Figure 4B**).

**Figure 5:**
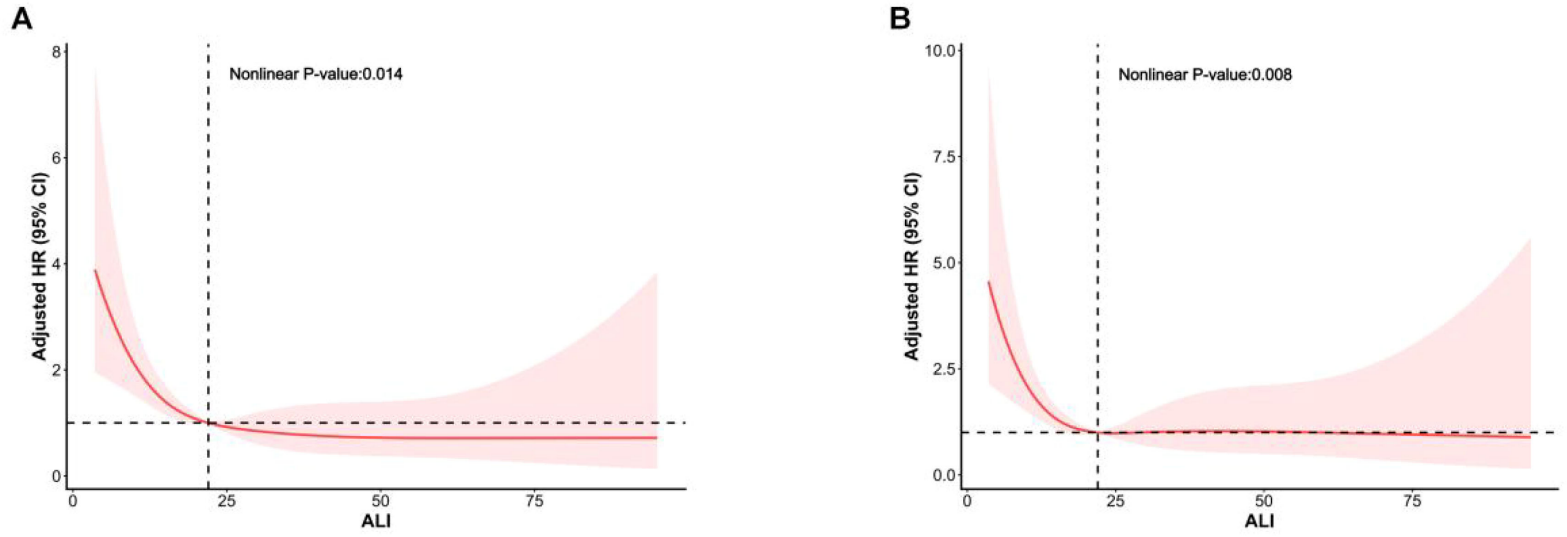
Restricted cubic spline (RCS) analysis of the non-linear associations between ALI and all-cause death **(A)** and cardiovascular death **(B)**.

Restricted cubic spline (RCS) models were employed to evaluate the dose-response relationship between continuous ALI and mortality risk. As illustrated in **Figure 4**, the analysis revealed a significant non-linear, L-shaped association of ALI with both all-cause mortality (P for non-linearity = 0.014; **Figure 5A**) and cardiovascular mortality (P for non-linearity = 0.008; **Figure 5B**). Specifically, below the critical threshold of 22, the adjusted risk of mortality increased precipitously as ALI levels declined.ROC curve analysis demonstrated that an ALI < 22 predicted long-term all-cause mortality with an AUC of 0.646 (95% CI: 0.595- 0.696, P < 0.001), yielding a sensitivity of 74.73% and a specificity of 54.37%.

### Incremental predictive value of ALI for all-cause death

We assessed the incremental predictive value of adding ALI to the GRACE score for all-cause mortality (**Table 4**). Adding ALI numerically improved the model’s C-index from 0.793 to 0.804. More importantly, it yielded statistically significant improvements in risk reclassification and overall discrimination, evidenced by a continuous net reclassification improvement of 0.228 (95% CI: 0.000–0.344, P = 0.047) and an integrated discrimination improvement of 0.027 (95% CI: 0.003–0.068, P = 0.020).

**Table 4:** The incremental predictive value of adding ALI to the GRACE score.

index	GRACE	GRACE + ALI	P
C-index	0.793	0.804	
Continuous NRI (95% CI)	Reference	0.228 (0.000-0.344)	0.047
IDI (95% CI)	Reference	0.027 (0.003-0.068)	0.020

GRACE, Global Registry of Acute Coronary Events; IDI, integrated discrimination improvement; NRI, net reclassification improvement.

### Subgroup analysis and sensitivity analysis

**Figure 6:**
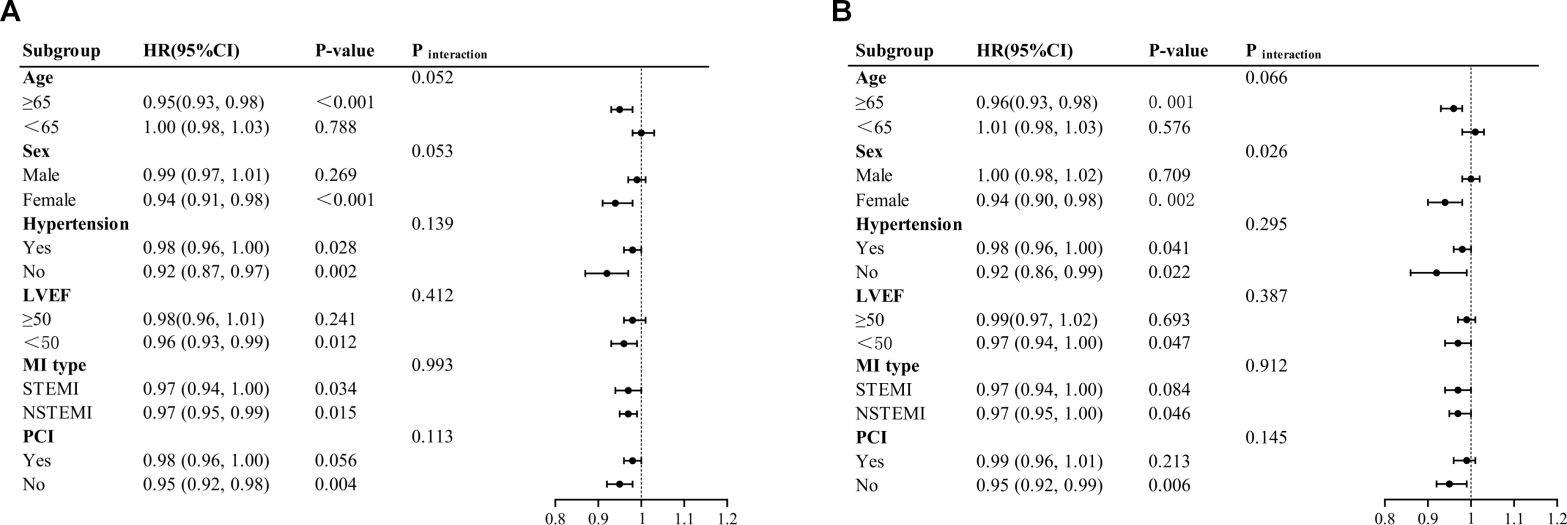
Subgroup analyses of the associations between ALI and all-cause death **(A)** and cardiovascular death **(B)**.

To further evaluate the risk stratification utility of ALI for the primary endpoints, subgroup analyses were performed by stratifying the study cohort according to sex, age, hypertension, LVEF, MI type, and PCI. Overall, the inverse association between ALI and both all-cause and cardiovascular mortality remained largely consistent across most clinical subgroups. Specifically, this protective association was particularly pronounced among patients who were elderly, female, without hypertension, and those not undergoing PCI (P < 0.05). Regarding all-cause mortality, none of the stratification variables exhibited significant interactions (all P for interaction > 0.05). Conversely, for cardiovascular mortality, a significant interaction with sex was detected (P for interaction = 0.026), indicating that the prognostic benefit of a higher ALI was markedly stronger in females (HR = 0.94, 95% CI: 0.90–0.98) than in males (**Figure 6**).

A sensitivity analysis was performed by incorporating medications as covariates (**Supplementary Table 3**). In the fully adjusted model, ALI remained significantly associated with reduced risks of all-cause (HR = 0.97, 95% CI: 0.95–0.99) and cardiovascular mortality (HR = 0.98, 95% CI: 0.96–1.00). Furthermore, categorical analysis by tertiles maintained a significant negative gradient. Compared with the T1 group, T2 and T3 exhibited substantially lower risks for all-cause death (HR = 0.41 and 0.35, respectively), and similar significant reductions were observed for cardiovascular death (HR = 0.38 and 0.44, respectively).

## Discussion

In this study of 571 AMI patients with diabetes, we demonstrated that lower admission ALI independently predicted increased long-term all-cause and cardiovascular mortality. Our analyses systematically verified that patients situated in the bottom ALI tertile had a significantly higher risk of both all-cause and cardiovascular fatalities relative to their counterparts in the uppermost tertile. Moreover, ALI provided significant incremental predictive value beyond the GRACE score, as reflected by significant NRI and IDI improvements. These findings establish ALI as a novel, readily accessible prognostic marker for this high-risk population.

Our findings are consistent with and extend existing literature that has explored the utility of ALI across distinct cardiovascular subsets. In patients with STEMI undergoing primary PCI, lower ALI independently predicted all-cause mortality (HR: 0.95, 95% CI: 0.92–0.97) and demonstrated superior discriminatory power over NLR alone (AUC: 0.732 vs. 0.685) (11). In MINOCA patients, lower ALI was an independent predictor of MACE (13),while in NSTEMI patients, it significantly correlated with higher SYNTAX scores, serving as an effective surrogate for coronary lesion complexity (17).This prognostic consistency extends across the spectrum of heart failure; lower ALI independently predicts both in-hospital and 90-day mortality in critically ill cohorts (where its addition to the GWTG-HF score significantly improves risk reclassification) (12), as well as long-term adverse outcomes in broader populations, including those with preserved ejection fraction (HFpEF) (18, 19). In broad population cohorts, elevated ALI demonstrated significant inverse associations with long-term all-cause and cardiovascular mortality among patients with T2DM (15), hypertension (20–22), metabolic dysfunction-associated steatotic liver disease (23), and CKM syndrome (24). Critically, the present study extends this evidence to a previously uninvestigated population—AMI patients with concomitant diabetes—and is the first to identify the specific prognostic significance of ALI within this high-risk diabetic-AMI comorbidity context.

Our prior work demonstrated that SII independently predicted long-term mortality specifically in AMI patients with diabetes but not in non-diabetics from the same registry, suggesting that the prognostic value of inflammation-based markers is more pronounced within the diabetic metabolic milieu. The present findings build upon this observation, showing that SII was an independent predictor of long-term mortality exclusively in diabetic AMI patients (HR = 2.90, 95% CI: 1.40–6.01) (9). The diabetic microenvironment amplifies ALI’s prognostic capacity through several converging mechanisms. Chronic hyperglycemia sustains persistent systemic inflammation and activates NF-κB signaling, priming neutrophils to overproduce superoxide and promoting neutrophil extracellular trap (NET) formation—a process pivotal to DM-related cardiovascular complications (25, 26). Concurrently, diabetic nephropathy accelerates urinary protein loss and hypoalbuminemia (27), while insulin resistance and lipid dysregulation alter the metabolic significance of BMI, such that even modest reductions may reflect disproportionate lean mass depletion (28). Collectively, these mechanisms alter all three ALI components in the diabetes-AMI state, explaining ALI’s particular sensitivity for mortality risk stratification in this population.

RCS analysis revealed significant non-linear, L-shaped associations between continuous ALI and both all-cause and cardiovascular mortality. An ALI value of 22 marked a critical threshold. Below this inflection point, mortality risk increased sharply with decreasing ALI, whereas the protective effect progressively attenuated at higher values. This pattern mirrors findings in diabetes and hypertensive populations (15, 21), suggesting a critical threshold below which the combined inflammatory and nutritional imbalances captured by ALI confer disproportionately elevated mortality risk, with the greatest potential benefit from risk-directed intervention concentrated at the lower end of the ALI distribution.However, caution is warranted when applying the threshold identified in this study to contemporary clinical practice. Our cohort data (2014–2018) predate the era of routine use of SGLT2 inhibitors and GLP-1 receptor agonists (29, 30). Given their pleiotropic effects, these two drug classes could theoretically modulate all components of the ALI: they reduce the NLR via anti-inflammatory pathways (31), mitigate urinary albumin loss, and alter BMI-related parameters (32, 33). Consequently, the widespread adoption of these novel glucose-lowering agents today may shift the overall distribution of ALI values among patients, potentially altering the optimal risk stratification threshold established in our study.

Although the addition of ALI to the traditional GRACE score yielded statistically significant improvements in the C-index and IDI, it must be acknowledged that the absolute improvement in model discrimination was modest (C-index: from 0.793 to 0.804). Furthermore, the lower bound of the confidence interval for the NRI touched zero (95% CI: 0.000–0.344, P = 0.047), indicating marginal significance. This pattern mirrors findings in critically ill HF patients, where adding ALI to the GWTG-HF score significantly improved both C-statistic and reclassification indices (NRI: 0.44; IDI: 0.03) (12), and in SA-AKI patients, where ALI provided incremental prognostic value beyond the SOFA score (34). Critically, the GRACE score primarily reflects hemodynamic and renal parameters but does not capture the composite inflammatory-nutritional-metabolic milieu; ALI, derived entirely from universally available admission data—BMI, serum albumin, and differential blood count—thus serves as a practical, cost-free complement rather than a replacement of existing risk tools, requiring no additional testing or cost (35).

Subgroup analysis revealed a significant sex interaction for ALI in cardiovascular mortality, with women showing a stronger protective effect. Initially, women tend to have more subcutaneous fat, influencing their metabolic and inflammatory profiles (36). Secondly, estrogen modulates women’s baseline inflammatory responses, making them more sensitive to changes post-menopause or during acute myocardial infarction (37). Ultimately,sex differences in liver albumin metabolism and nutritional reserves also contribute under ischemic stress (38).

Low ALI predicts adverse cardiovascular outcomes through integrated inflammatory, nutritional, and metabolic pathways. Elevated NLR reflects persistent systemic inflammation, where neutrophil-derived cytokines promote endothelial dysfunction, reduce nitric oxide bioavailability, and enhance plaque vulnerability and thrombosis (39, 40). Decreased serum albumin indicates malnutrition and oxidative stress, both of which impair endothelial repair capacity and exacerbate vascular inflammation, facilitating atherosclerosis progression and increasing risk of myocardial infarction and coronary flow abnormalities (41). Meanwhile, abnormal BMI, particularly visceral adiposity, sustains adipokine-mediated inflammation and insulin resistance, accelerating macrovascular damage (42). These components converge on a unified axis of endothelial dysfunction–chronic inflammation–metabolic disturbance, ultimately driving atherosclerotic instability (43–45). The persistent inflammation reflected by a low ALI not only triggers plaque instability but also exerts profound downstream effects on post-infarction myocardial healing. As noted by Trimarchi et al (46) unresolved inflammation following a myocardial infarction is a key driver in the progression of chronic heart failure. Given that diabetes intrinsically impairs myocardial repair, this sustained systemic inflammation is particularly detrimental in diabetic patients with AMI, accelerating adverse ventricular remodeling and ultimately leading to heart failure. Moreover, given the significant fluctuations of ALI components during the acute phase of AMI—where myocardial necrosis triggers rapid NLR spikes, and acute-phase reactions or edema transiently alter albumin and BMI—admission ALI is best interpreted as a composite metric integrating both the chronic metabolic-nutritional baseline and acute physiological stress (4, 47).

Several limitations of this study warrant acknowledgment. First, the retrospective, single-center design of the NOAFCAMI-SH registry limits causal inference and restricts the generalizability of findings. Second, as ALI was assessed solely at admission, its dynamic trajectory remains unexplored; thus, whether improving ALI via nutritional or anti-inflammatory interventions yields tangible clinical benefits requires prospective validation. Third, despite multivariable adjustment, residual confounding from unmeasured variables—including dietary intake, medication adherence, glycemic trajectory, and baseline nutritional status or chronic wasting diseases—can not be fully excluded. Finally, our cohort is predominantly East Asian. Given their lower cardiovascular risk thresholds for BMI compared to Westerners, and with BMI being core to ALI, our cut-off values cannot be directly extrapolated to non-Asian populations.

## Conclusion

In conclusion, a lower admission ALI is independently associated with all-cause and cardiovascular mortality in diabetic patients with AMI. As a simple, cost-effective, and reproducible composite marker, ALI offers a practical tool for early risk stratification and individualized clinical management in this high-risk population, though prospective validation remains warranted.

## Data Availability

The data analyzed in this study is subject to the following licenses/restrictions: The datasets used and/or analyzed during this study are available from the corresponding author on reasonable request. Requests to access these datasets should be directed to lihongqiang1984@163.com.

## Supplementary material

Supplementary Figure 1 Selection of risk factors for all-cause mortality using the LASSO regression model. **(A)** A total of 22 variables were included in the LASSO binary logistic regression analysis. **(B)** Two dotted lines indicate the optimal values selected by the minimum criteria (λ.min) and the 1-SE criteria (λ.1se) on the LASSO coefficient profiles. The selected variables include age, heart rate, creatinine, PCI, ACEI/ARB, β-blockers, Killip class, and glucose.

Supplementary Table 1 Univariable Cox regression analysis of the association of ALI with all-cause mortality.

Supplementary Table 2 Univariable Cox regression analysis of the association of ALI with cardiac mortality.

Supplementary Table 3 Sensitivity analysis of the association of ALI with all-cause and cardiac death.
