# Supplementary material for: Prognostic significance of the advanced lung cancer inflammation index for long-term mortality in patients with acute myocardial infarction and diabetes
Source: Front Endocrinol (Lausanne). 2026 Jul 6;17:1876399. doi: 10.3389/fendo.2026.1876399 (PMC13381637; doi:10.3389/fendo.2026.1876399)
Supplement: Supplementary Figure 1 — Selection of risk factors for all-cause mortality using the LASSO regression model. (A) A total of 22 variables were included in the LASSO binary logistic regression analysis. (B) Two dotted lines indicate the optimal values selected by the minimum criteria (λ.min) and the 1-SE criteria (λ.1se) on the LASSO coefficient profiles. The selected variables include age, heart rate, creatinine, PCI, ACEI/ARB, β-blockers, Killip class, and glucose. [file SupplementaryFile1.pdf]

Table S1 Univariable Cox regression analysis of the association of ALI with all-cause mortality

Variable	HR (95%CI)	P-value
Age	1.06 (1.04, 1.08)	<0.001
Male	1.73 (1.08, 2.75)	0.022
Current smoking	0.42 (0.26, 0.71)	0.001
Hypertension	2.44 (1.32, 4.52)	0.005
STEMI	0.82 (0.52, 1.28)	0.380
Killip class＞1	3.33(2.08, 5.32)	<0.001
PCI	0.19 (0.12, 0.31)	<0.001
Peak TnT	1.06 (1.00, 1.12)	0.037
HbA1c	0.91 (0.79, 1.04)	0.174
Creatinine	1.00 (1.00, 1.00)	<0.001
ALI	0.96 (0.94, 0.98)	<0.001

Abbreviations： STEMI: ST-elevation myocardial infarction.

Table S2 Univariable Cox regression analysis of the association of ALI with cardiac mortality

Variable	HR (95%CI)	P-value
Age	1.06 (1.04, 1.08)	<0.001
Male	1.61 (1.05, 2.47)	0.030
Current smoking	0.41 (0.25, 0.66)	<0.001
Hypertension	1.74 (1.05, 2.88)	0.032
STEMI	0.75 (0.50, 1.13)	0.175
Killip class＞1	3.30(2.14, 5.08)	<0.001
PCI	0.24 (0.16, 0.36)	<0.001
Peak TnT	1.04 (0.99, 1.10)	0.156
HbA1c	0.96 (0.84, 1.08)	0.470
Creatinine	1.00 (1.00, 1.00)	<0.001
ALI	0.96 (0.94, 0.97)	<0.001

Abbreviations： STEMI: ST-elevation myocardial infarction.

Table S3 Sensitivity analysis of the association of ALI with all-cause and cardiac death

Variable	Model 1		Model 2		Model 3	
	HR (95%CI)	P-value	HR (95%CI)	P-value	HR (95%CI)	P-value
All-cause death						
ALI	0.96 (0.94, 0.97)	<0.001	0.96 (0.94, 0.98)	<0.001	0.97 (0.95, 0.99)	0.002
ALI tertiles						
T1	1.00 (Ref)		1.00 (Ref)		1.00 (Ref)	
T2	0.35 (0.22, 0.58)	<0.001	0.36 (0.22, 0.60)	<0.001	0.41 (0.24, 0.71)	0.001
T3	0.24 (0.13, 0.42)	<0.001	0.29 (0.16, 0.53)	<0.001	0.35 (0.19, 0.66)	0.001
P for trend	0.46 (0.34, 0.61)	<0.001	0.50 (0.37, 0.67)	<0.001	0.56 (0.41, 0.77)	<0.001
Cardiac death						
ALI	0.96 (0.94, 0.98)	<0.001	0.96 (0.94, 0.98)	<0.001	0.98 (0.96, 1.00)	0.017
ALI tertiles						
T1	1.00 (Ref)		1.00 (Ref)		1.00 (Ref)	
T2	0.32 (0.18, 0.56)	<0.001	0.33 (0.19, 0.58)	<0.001	0.38 (0.20, 0.69)	0.002
T3	0.27 (0.15, 0.49)	<0.001	0.33 (0.18, 0.61)	<0.001	0.44 (0.23, 0.85)	0.014
P for trend	0.47 (0.35, 0.65)	<0.001	0.51 (0.37, 0.71)	<0.001	0.60 (0.43, 0.85)	0.004

Model 1: Unadjusted.

Model 2: Adjusted for age and gender.

Model 3: Adjusted for age, gender, current smoking, hypertension, STEMI, Killip class, PCI, peak TnT, HbA1c, creatinine, aspirin, ACEI/ARBs,β-blockers and stains.

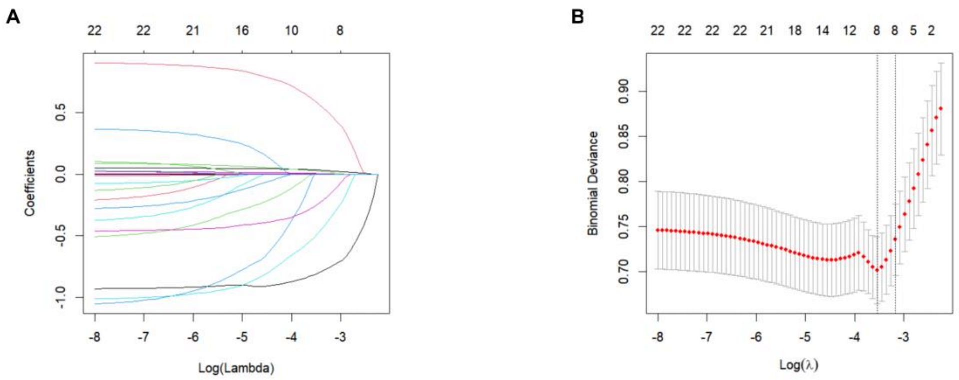

Figure S1 Selection of risk factors for all-cause mortality using the LASSO regression model. (A) A total of 22 variables were included in the LASSO binary logistic regression analysis. (B) Two dotted lines indicate the optimal values selected by the minimum criteria (λ.min) and the 1-SE criteria (λ.1se) on the LASSO coefficient profiles. The selected variables include age, heart rate, creatinine, PCI, ACEI/ARB, β-blockers, Killip class, and glucose.
